# Laparoscopic dismembered pyeloplasty for ureteropelvic junction obstruction in children: a single-surgeon experience

**DOI:** 10.3389/fped.2026.1735651

**Published:** 2026-04-01

**Authors:** Thuy Nguyen Thi Mai, Thao Nguyen Phuong

**Affiliations:** 1 Department of Urology, Vietnam National Children's Hospital, Hanoi, Vietnam; 2 Department of Surgery, Hanoi Medical University, Hanoi, Vietnam

**Keywords:** Anderson–Hynes, laparoscopic dismembered pyeloplasty, transabdominal suspension technique, transperitoneal, ureteropelvic junction obstruction

## Abstract

**Objective:**

The objective of this work is to evaluate the safety and short-term outcomes of laparoscopic dismembered pyeloplasty (LDP) for ureteropelvic junction obstruction (UPJO) in children, based on a single-surgeon experience using a transabdominal suspension technique.

**Methods:**

We retrospectively analyzed children with UPJO between January 2023 and October 2024 who underwent transperitoneal LDP with a transabdominal suspension maneuver performed by a single surgeon. The diagnosis was confirmed by ultrasonography and magnetic resonance urography. The outcomes were operative time, complications, hospital stay, and postoperative hydronephrosis reduction, assessed by ultrasonography at least 3 months after the operation.

**Results:**

A total of 60 children with a mean age of 58.5 months (range: 6–168 months) underwent surgery during the study period; of these, 47 were boys. The mean operative time was 140 min (range: 120–200 min). No open conversions or major complications occurred. The mean hospital stay was 3.5 days. The median pelvic diameter decreased from 35.6 mm (range: 25–50 mm) preoperatively to 13.5 mm (range: 8–22 mm) postoperatively (*p* < 0.05). All patients were free from symptoms with reduced hydronephrosis during the follow-up period.

**Conclusions:**

Transperitoneal laparoscopic dismembered pyeloplasty is a safe and effective option in the treatment of UPJO, and a transabdominal suspension technique may facilitate the procedure.

## Introduction

Ureteropelvic junction obstruction (UPJO) is the most prevalent cause of obstructive uropathy in children, potentially leading to renal impairment if untreated. The open Anderson–Hynes pyeloplasty has long been considered the gold standard, achieving success rates of over 90% (1–3). Minimally invasive approaches such as laparoscopic and robot-assisted techniques have emerged as viable options, offering lower morbidity and faster recovery. Various laparoscopic techniques through transperitoneal and retroperitoneal approaches have been reported (4, 5). Robot-assisted pyeloplasty is now being offered at some centers and it has demonstrated good efficacy; however, its high cost and large instrument size limit its availability in low-income countries (6, 7). This study reports our single-surgeon initial experience with transperitoneal laparoscopic dismembered pyeloplasty (LDP) in children, assessing its safety and short-term efficacy. We also describe a technical modification called the “transabdominal suspension technique,” designed to facilitate anastomosis in the limited working space of pediatric patients.

## Materials and methods

### Study design

This retrospective study included children with UPJO who underwent LDP performed by a single surgeon between January 2023 and October 2024.

### Inclusion criteria

Eligible patients included children with UPJO and a functioning kidney. UPJO was diagnosed by renal ultrasonography and magnetic resonance urography (MRU), defined as an anteroposterior pelvic diameter (APD) ≥20 mm with progressive dilation. Clinical manifestations of UPJO were a prenatal diagnosis of urinary tract dilation with progression on postnatal follow-up and clinical symptoms such as intermittent flank pain, fever, or abdomen lump. Tc-99 m DTPA renography was indicated in selected cases. Because nuclear renography was not widely available at our facility, it was indicated only for those children who had an APD ≥40 mm and renal cortical thickness <4 mm. Those with a smaller APD and thick renal cortex on MRU did not undergo routine renography.

### Exclusion criteria

Patients who had undergone prior renal surgery, had concurrent urinary tract anomalies, or who had active infection (treated with antibiotics until negative urine culture results were obtained) were excluded from the study. Patients with non-functioning kidneys were also excluded.

### Variables

Preoperative characteristics were age, gender, and preoperative APD measured on MRU. Immediate surgical results were operative time, conversion to open surgery, complications [classified using the Clavien–Dindo scale (8), with complications of grade ≥3A considered severe], and length of hospital stay. Short-term surgical results were APD at the most recent follow-up ultrasonography and symptoms relevant to UPJO, with short-term improvement defined as a marked reduction in APD and the absence of symptoms.

### Surgical technique

The operative procedure followed Anderson–Hynes principles through a transperitoneal approach. The procedure was carried out under general anesthesia; a 4-Fr double-J stent was inserted into the affected ureter by cystoscopy before incision. Patients were then placed in the flank position, with a nasogastric tube and a Foley catheter inserted. Three trocars (3 mm for children under 2 years, 5 mm for older children) were inserted: one umbilical port for the 30° camera and two working ports. Intra-abdominal pressure was maintained at 10–12 mmHg.

**Figure 1:**
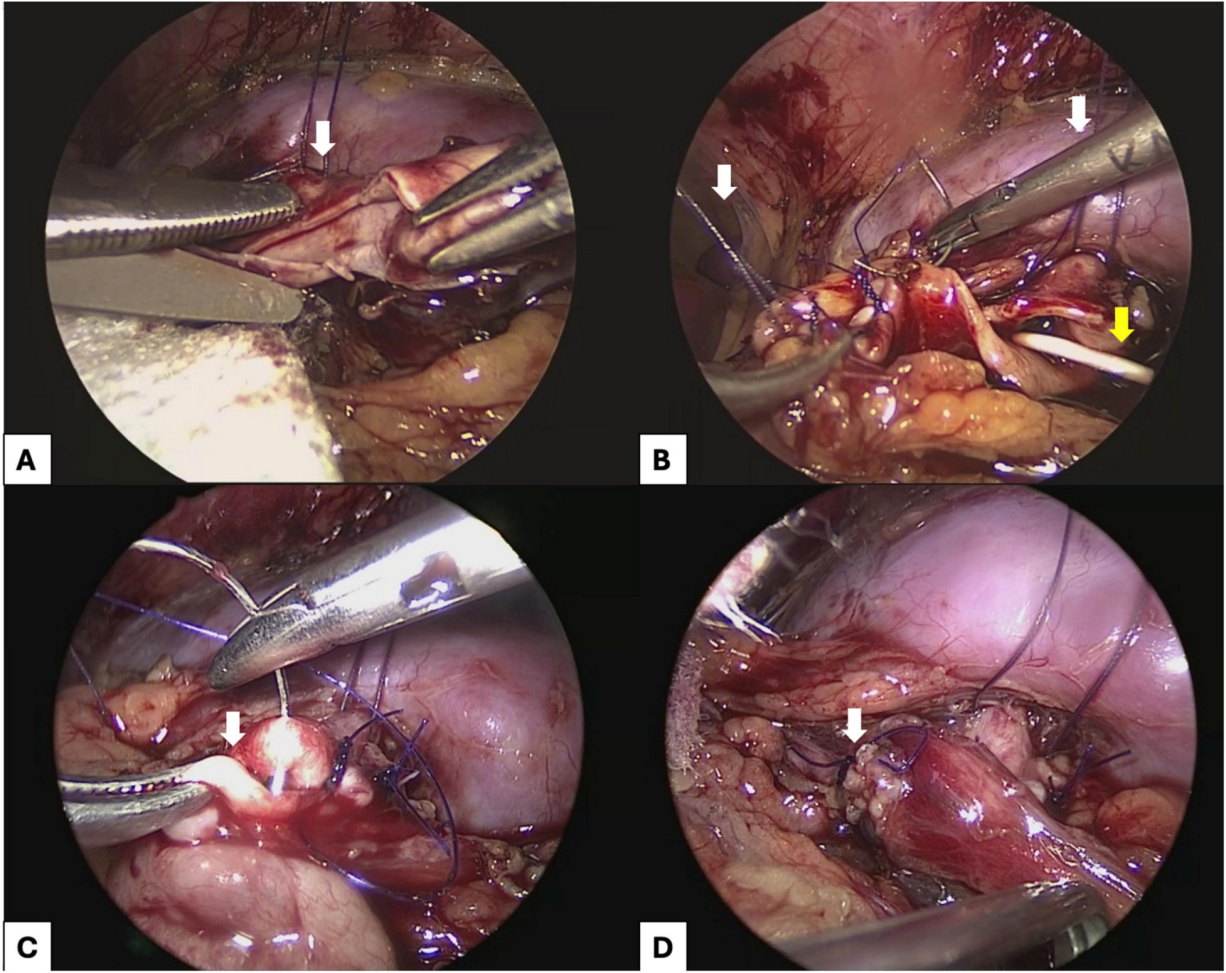
Surgical technique of transabdominal laparoscopic dismembered pyeloplasty, with a transabdominal suspension technique. **(A)** Excision of the excess renal pelvis (white arrow). **(B)** Reduction pyeloplasty with polydioxanone (PDS) 5-0 sutures, with a previously placed intraluminal double-J stent (yellow arrow). Note the transabdominal stay suture (white arrows) suspending the ureter to facilitate anastomosis. **(C)** Reanastomosis between the pelvis and the ureter (white arrow). **(D)** Completed anastomosis (white arrow).

The anterior renal fascia was transected longitudinally, and the lower pole of the kidney was identified. The anterior surface of the kidney was also exposed. Then, the dilated renal pelvis, the ureteropelvic junction (UPJ), and the upper ureter were completely dissected. When crossing vessels were present over the UPJ, they were mobilized and separated from the urinary tract. Two stay sutures (Vicryl 5-0 for children under 2 years old, Vicryl 4-0 for older children) were inserted through the abdominal wall for performing a suspension maneuver at the renal pelvis—this was referred to as our “transabdominal suspension technique.” With gentle traction, these stay sutures completely exposed the renal pelvis, UPJ, and ureter. The renal pelvis was dismembered, with a resection of the proximal ureter and stenotic segment, followed by a lateral spatulation of the ureter and inferior extension through the narrowed portion for approximately 2 cm. The renal pelvis was reduced by removing the dilated segment. If crossing vessels were present, the UPJ was dismembered, and the ureter and pelvis were transposed and anastomosed ventrally to the crossing vessels. The pelvis was closed with interrupted 5-0 polydiaxone (PDS) sutures. Renal pelvis reduction was also routinely performed. Anastomosis was then completed using running 5-0 PDS sutures, starting from the furthest point of the spatulated ureter and proceeding to the lower edge of the pelvis (Figure 1).

At the conclusion of the procedure, a 10-Fr plastic drain was placed through one of the working ports into the pararenal space. The Foley catheter was left in place for 48 h. The double-J stent was removed after 6 weeks with no prophylaxis antibiotics.

### Statistical analysis

Data were analyzed using IBM SPSS Statistics for Windows, version 20 (IBM Corp., Armonk, NY, USA). Continuous variables were expressed as mean/median (minimum–maximum), while discrete variables were expressed as number of cases (percentage). Preoperative and postoperative APDs were compared using a paired *t*-test, with *p* < 0.05 considered statistically significant.

### Ethics consideration

All patient information was anonymized and kept confidential throughout the study. No personal identifiers were disclosed in any form. Ethical approval for this study was waived by the Institutional Review Board of Vietnam National Children's Hospital (IRB-VN01037/IRB00011976/FWA00028418) because of its retrospective nature. Written inform consent was obtained from the legal guardians of the patients prior to data collection.

## Results

### Patient characteristics

All 60 procedures were completed laparoscopically without conversion or major perioperative complications, following the principles of the open Anderson–Hynes procedure. Participants comprised 47 boys and 13 girls, aged 6–168 months, with a mean age of 58.5 months. The median preoperative renal APD was 35.6 mm (range: 25–50 mm).

### Operative results

The average operative time (excluding cystoscopy) was 140 min (range: 120–200 min). Seven cases with crossing vessels were recorded. Patients resumed a liquid diet 6 h postoperatively, advancing to full feeding within 1–2 days. Pain was managed with paracetamol, and hospital stay averaged 3.5 days (range: 3–5 days). The double-J stent was removed at 6 weeks, followed by ultrasonography.

### Postoperative follow-up

The mean follow-up duration was 8.5 months (range: 3–18 months). The median renal pelvic anterior-posterior diameter at ultrasonography significantly decreased from 35.6 mm (25–50 mm) preoperatively to 13.5 mm (range: 8.0–22.0 mm) postoperatively (*p* < 0.05) (Table 1).

**Table 1:** Patient characteristics, operative results, and postoperative follow-up.

Characteristic	Value
Number of patients	60
Age (months)	58.5 (6–168)^a^
Gender (boys/girls)	47/13
Preoperative pelvic diameter on MRU (mm)	35.6 (25–50)^a^
Operative time (minutes)	140 (120–200)^a^
Presence of crossing vessels (percentage)	7 (11.7%)
Number of cases requiring conversion to open surgery number (percentage)	0 (0%)
Number of cases with intermediate complication (percentage)	0 (0%)
Length of hospital stay (days)	3.5 (3–5)^a^
Follow-up time (months)	8.5 (3–18)^a^
Postoperative pelvic diameter on ultrasonography (mm)	13.5 (8–22)^b^
Short-term improvement (percentage)	60 (100%)^b^

^a^ Data presented as mean (minimum–maximum) values.

^b^ Data presented as median (minimum–maximum) values.

During the postoperative period, 5 patients experienced fever following the double-J stent indwelling, but their urine culture results were negative, and therefore, urinary tract infection (UTI) was ruled out. During the mean follow-up period of 8.5 months, no reintervention was needed. However, there were 8 patients who had a postoperative APD >20 mm. Renography was indicated for these patients. There was no evidence of anastomotic stricture nor persistent obstruction that required reintervention.

## Discussion

UPJO is a prevalent pediatric urological condition that may impair renal function and lead to parenchymal atrophy if untreated (1). Various treatment options, including endopyeloplasty, endopyelotomy, and pyeloplasty, are available, with Anderson–Hynes pyeloplasty remaining the gold standard, achieving success rates above 90% (2, 3). Advancements in minimally invasive techniques and instrumentation have facilitated the adoption of antegrade and retrograde endopyelotomies for UPJO management. Nevertheless, the success rates associated with pyelotomy are typically inferior to those of Anderson–Hynes pyeloplasty (9, 10). Laparoscopic pyeloplasty, introduced in the 1990s, has emerged as a minimally invasive alternative to UPJO treatment. Following its initial developmental phase, it now yields success rates comparable to, and occasionally surpassing, those of open surgery (2–5). The laparoscopic Anderson–Hynes procedure can be executed via two primary routes: transperitoneal or retroperitoneal.

In the retroperitoneal approach, the operative field often appears less distinct, and the confined retroperitoneal space in pediatric patients is presumed to hinder laparoscopic maneuverability. Moreover, the retroperitoneal approach is rendered more challenging when UPJO is secondary to crossing vessels. On the other hand, the transperitoneal approach can disturb other intraperitoneal organs, and urine leakage caused by the operation may lead to some serious consequences. The choice of approach depends primarily on the preference of the surgeon. Both retroperitoneal and transperitoneal approaches yield similar results in terms of postoperative complications, conversion rates, and recurrence, but retroperitoneal laparoscopy may offer potential benefit for patients by enabling faster recovery as it can reduce the time of postoperative drainage (4, 5, 11). At present, ascertaining superiority between the two approaches remains controversial, as retroperitoneal laparoscopy demands longer operating times and a steeper learning curve. Thus, at present, surgeons should select the approach best aligned with their experience.

The mean operative time in our series was 140 min (range: 120–200 min), which is comparable to other cohorts published in the literature (2, 5, 7). From our experience, a preoperative insertion of a double-J stent under cystoscopy facilitated the identification and dissection of the renal pelvic and the upper part of the ureter. It also allowed the surgeon to assess the ureteral orifices. There were concerns that preoperative stent placement might make subsequent dissection and anastomosis more challenging; however, we did not experience any difficulty. The double-J stent helped in the drainage of the pelvis and the ureter, which facilitated subsequent dissection and resection. Our transabdominal suspension technique was used to suspend and apply traction to the renal pelvis for severance of the stenotic segment of the ureter. The technique helped stabilize the segment to be resected, ensuring a stable position, reducing suturing time, and possibly preventing torsion of the anastomosis. This was also applicable in the cases of UPJO secondary to crossing vessels—this technique allowed for better visualization and stabilization when transposing the ureter. We believe that this maneuver allowed us to significantly reduce the operative time and facilitate the suturing process. It is particularly suitable in small children who have limited abdominal space that restricts the manipulation of laparoscopic instruments. Moreover, by suspending and stabilizing the ureter and the pelvis, this technique might help surgeons with less experience to perform the procedure, especially in the absence of more stable robotic instruments. Robot-assisted surgery has been used routinely in some centers with good benefits, particularly in terms of shortening the learning curve of surgeons. However, in many resource-constrained countries, robotic surgery is not widely available. In such cases, our technique may help surgeons with less experience to perform this operation.

This study is limited by its retrospective design and single-center, single-surgeon cohort, potentially introducing selection bias and limiting generalizability. Reliance on morphological outcomes without routine functional imaging (e.g., renography) may overestimate success. Furthermore, the absence of a control group makes it difficult to infer the effectiveness of our modification compared with the conventional technique. Prospective and comparative studies with extended monitoring might be needed to address the limitations of this study.

## Conclusion

Transperitoneal laparoscopic dismembered pyeloplasty is a safe and effective option for the treatment of ureteropelvic junction obstruction in children. The addition of a transabdominal suspension technique, combined with preoperative double-J stent placement, may facilitate the procedure, particularly in resource-constrained countries where robotic-assisted surgery capabilities are limited.

## Data Availability

The raw data supporting the conclusions of this article will be made available by the authors without undue reservation.
